# Necrotizing herpetic retinopathy in an immune-compromised pediatric patient with minimal signs of inflammation: case report

**DOI:** 10.1186/s12886-016-0253-x

**Published:** 2016-06-08

**Authors:** Soon Il Choi, Jae Ryun Kim, Ho Ra

**Affiliations:** Department of Ophthalmology and Visual Science, College of Medicine, The Catholic University of Korea, Bucheon St. Mary’s Hospital, #327 Sosa-ro, Wonmi-gu, Bucheon 420-717 Korea

**Keywords:** Acute retinal necrosis, Necrotizing herpetic retinopathy

## Abstract

**Background:**

To report a case of necrotizing herpetic retinopathy(NHR) in an immuno-compromised pediatric patient.

**Case presentation:**

An 11-year-old boy presented with a minimal ocular foreign-body sensation and peripheral visual-field defect, as well as mild upper respiratory symptoms. He had undergone the Fontan operation for a ventricular septal defect and single ventricle during infancy, and had been taking oral steroids for 1 year immediately prior to his presentation to treat protein-losing enteropathy. Initially, a case of either cytomegalovirus (CMV) retinitis or acute retinal necrosis (ARN) was suspected, and an intravenous course of ganciclovir and acyclovir was therefore initiated. During treatment, varicella-zoster virus (VZV) was detected in the anterior chamber, and ARN was confirmed when both serum and aqueous humor were found by polymerase chain reaction (PCR) to be positive for VZV DNA. A peripheral retinal break and detachment developed after medical treatment, and a vitrectomy was performed.

**Conclusions:**

Typically, ARN is found in both healthy individuals and subclinically immuno-compromised patients of any age. CMV retinitis is somewhat more typical for immuno-compromised patients. Herein, we report a case of NHR in a pediatric patient with poor general condition and showing minimal signs of inflammation.

## Background

Acute retinal necrosis (ARN) is diagnosed clinically using the American Uveitis Society’s criteria for ocular manifestations (1994). Microbial examinations of the virulent agent and laboratory findings regarding systemic status play only a supporting role. Therefore, the ophthalmologist’s clinical diagnosis is very important to ensure early identification and treatment of the condition. In this case, the patient presented with minimal ocular inflammation and visual field defect. Moreover, the patient had undergone the Fontan operation for a cardiac anomaly, and had been taking oral steroids for 1 year immediately prior to presentation to treat protein-losing enteropathy (PLE). For these reasons, retinitis, caused by either ARN or cytomegalovirus (CMV), was initially suspected, and acyclovir and ganciclovir were therefore intravenously administered. However, the anterior chamber was later found by polymerase chain reaction (PCR) to be positive for varicella zoster virus DNA. This, combined with the specific retinal lesions, indicated that a diagnosis of necrotizing herpetic retinopathy(NHR) was more fitting. Our case demonstrates that NHR should not be ruled out in situations with manifestations atypical to the disease. To our knowledge, this is the first described case of a necrotizing herpetic retinopathy in a PLE patient.

## Case presentation

An 11-year-old boy presented, complaining of a floater and peripheral visual field defects in his right eye that had persisted for 1 week. He had also had a mild upper respiratory infection for 2 weeks, but no characteristic skin lesions. He had undergone Fontan surgery for ventricular septal defect during infancy, and had been taking oral prednisolone (7.5 mg daily) for protein-losing enteropathy (PLE) for 1 year prior to presentation. His height was 153 cm, and his weight was 32 kg (Body Mass Index = 13.9).

**Fig. 1:**
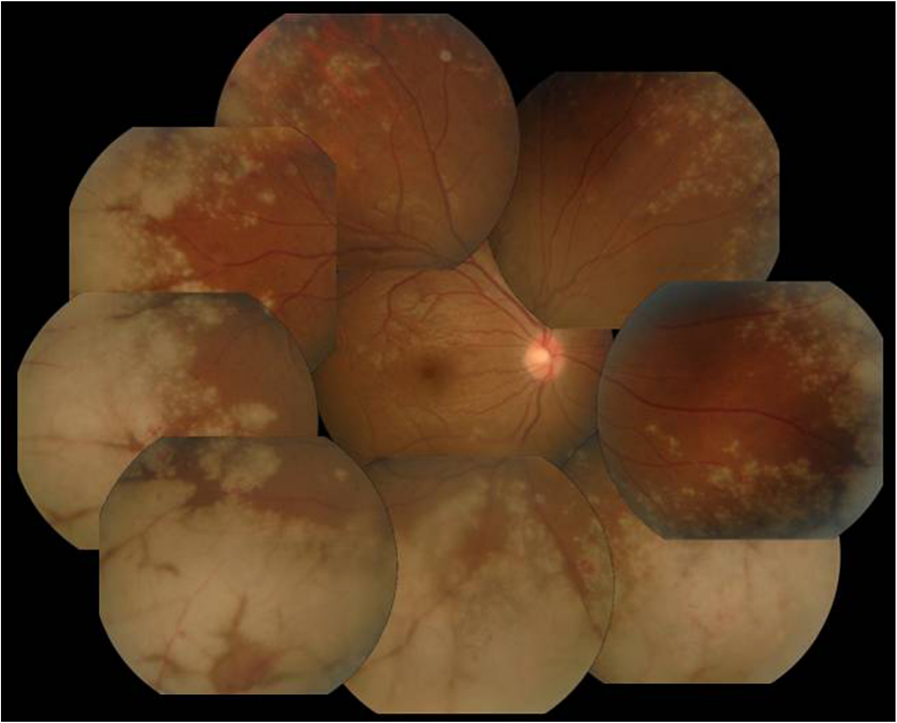
Discrete, whitish retinal infiltration affecting the far periphery of the retina in all quadrants of the right eye

**Fig. 2:**
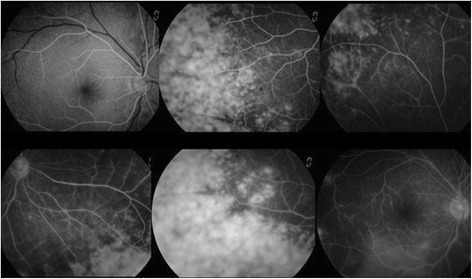
Fluorescein angiography revealed late leakage from the retinal vessels in the right eye

Ophthalmologic examination revealed a best-corrected visual acuity (BCVA) of 20/20 in both eyes, and there was no afferent pupillary defect. The respective intraocular pressures were 21 mmHg in the right eye and 18 mmHg in the left eye. Minimal conjunctival hyperemia was present, and there were only a few anterior chamber cells in the right eye. Funduscopy revealed optic disc peripheral hyperemia that covered 360° of the retinal circumference, as well as a discrete, whitish retinal infiltration affecting the far periphery of the retina in all quadrants of the right eye (Fig. 1). There was no obvious evidence of vitritis. Slit-lamp examination of the left eye yielded no remarkable results. Fluorescein angiography revealed late leakage from the retinal vessels in the right eye (Fig. 2).

The patient’s total lymphocyte count was diminished in that his total white blood cell count was 10.79 × 10^9^/L, of which lymphocytes composed 5 % (reference range 20–44 %), whereby the absolute lymphocyte count was 540 cells/μl at presentation. Excluding the lymphocyte count, there were no laboratory abnormalities as regards hematologic parameters. Serological tests were positive for both varicella-zoster virus (VZV) IgG and cytomegalovirus (CMV) IgG, whereas the tests were negative for herpes-simplex virus (HSV) IgG. IgM titers for HSV, VZV, and CMV, which can be positive in cases of recent infection, were all negative. Anterior chamber paracentesis was performed, and the aqueous humor was tested, using polymerase chain reaction (PCR), for VZV, HSV-1 and -2, and CMV DNA.

Considering the clinical features and immunological status of the patient, we initially suspected CMV retinitis, acute retinal necrosis (ARN), or progressive outer retinal necrosis (PORN). We resolved to administer intravenous (IV) ganciclovir (5 mg/kg, twice daily) and IV acyclovir (12 mg/kg, 3 times daily) simultaneously. Potential systemic side effects of the drugs were carefully monitored, and oral prednisolone was continued.

**Fig. 3:**
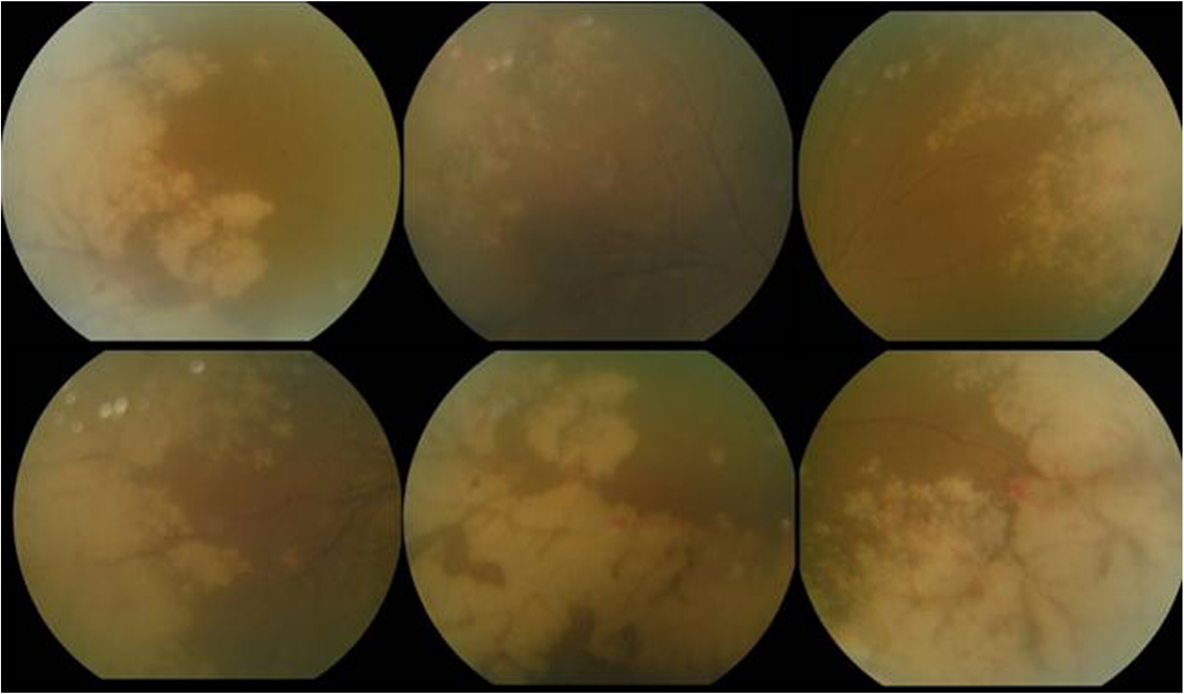
Retinal infiltration and hemorrhaging had progressed, and vitreous hemorrhaging and opacity had newly developed in the right eye at 3 days after administering intravenous (IV) ganciclovir and IV acyclovir simultaneously

By day 3 of therapy, retinal infiltration and hemorrhaging had progressed, and vitreous hemorrhaging and opacity had newly developed in the right eye (Fig. 3). At this time, PCR of an aqueous humor sample was positive for VZV, but negative for HSV-1 and -2, and CMV. IV ganciclovir was stopped, and an increased dose of IV acyclovir was initiated (15 mg/kg, 3 times daily).

**Fig. 4:**
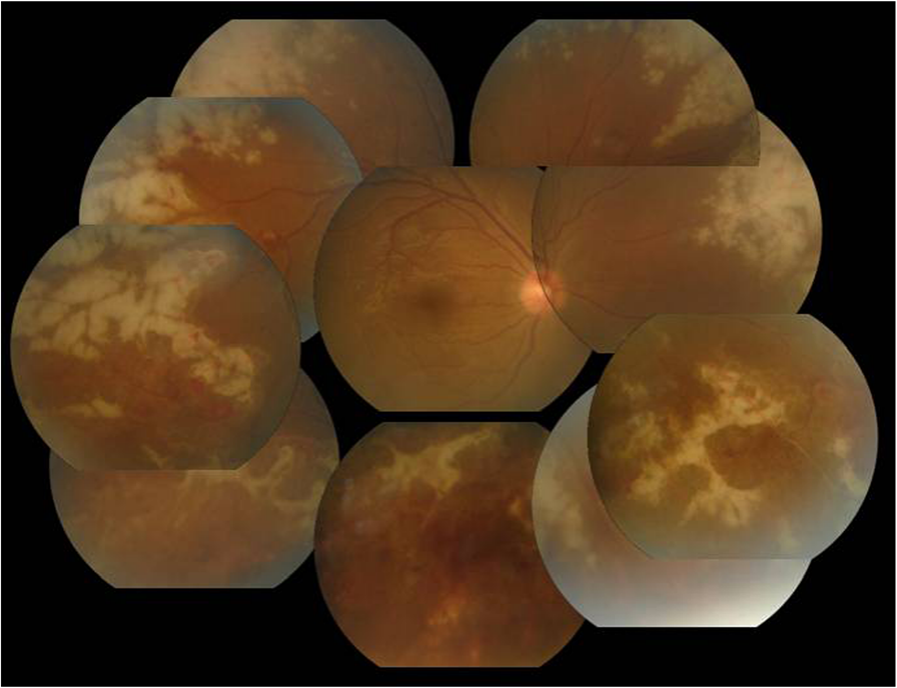
Retinal infiltration and retinal hemorrhaging had substantially decreased at 3 days after IV ganciclovir was stopped, and an increased dose of IV acyclovir was initiated

**Fig. 5:**
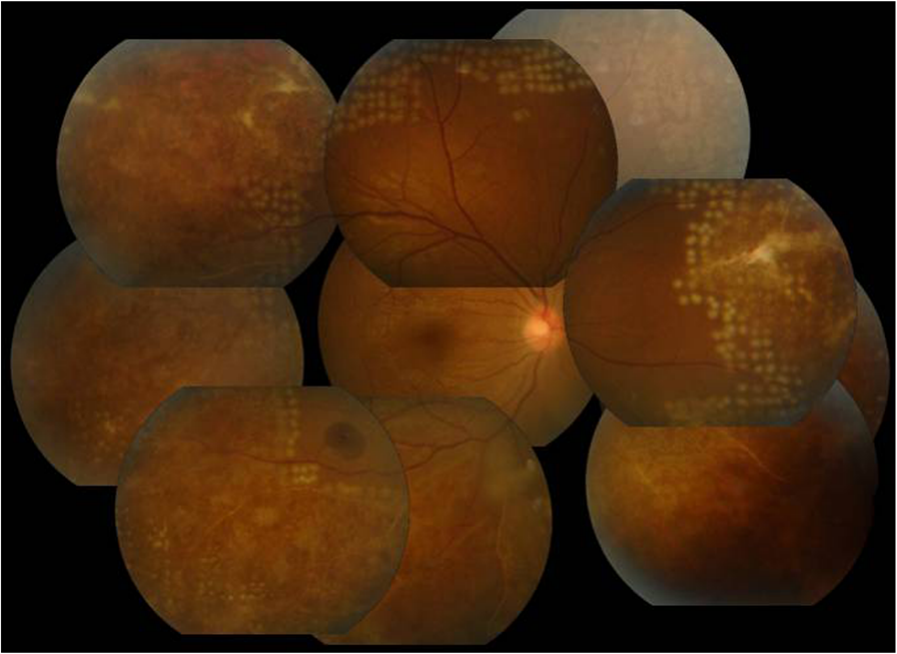
Barrier laser was applied after focal atrophy with traction membrane was manifested

After 3 days of the above treatment, retinal infiltration and retinal hemorrhaging had substantially decreased (Fig. 4). However, focal atrophy with traction membrane manifested 2 days later, and barrier laser was applied (Fig. 5).

The patient was treated using oral valacyclovir (1000 mg, 3 times daily) after completing the 2-week course of IV acyclovir. The same dose of oral prednisolone was maintained throughout.

Two weeks later, the patient had developed a rhegmatogenous retinal detachment, in spite of the laser photocoagulation, and a pars plana vitrectomy with silicone oil filling was performed. The retinal reattachment surgery was successful, and the final BCVA in the right eye was 20/40. The left eye remained uninvolved.

## Conclusions

ARN is a rare, but vision-threatening disease that involves retinal necrosis and subsequent detachment. The condition was initially described by Urayama in 1971. Typical ARN is characterized by extensive, rapidly-progressing, full-thickness retinal necrosis and associated severe vitreous or anterior chamber inflammation [1]. PORN is a similar disease entity; it is characterized by necrosis of the retina involving the posterior pole early in the course of disease, and virtually no associated vitreous or anterior chamber inflammation [2].

However, several cases of mild ARN have been reported that were associated with discrete immune dysfunctions; these had several features of PORN, which is usually seen in more immuno-compromised patients [3]. Guex-Crosier et al. suggested that the degree of immune impairment determines the type and severity of the disease, with intermediary clinical forms existing between the classical forms of ARN and PORN. In addition, they proposed using the term “necrotizing herpetic retinopathy” (NHR)—a denomination proposed by the American Uveitis Society as an umbrella term covering the whole spectrum: from ARN on the one side to PORN on the other [4, 5].

NHRs are caused by VZV, HSV-1 and 2, CMV, and Epstein-Barr virus (rarely). As stated above, the spectrum of clinical presentations may depend on the host’s immunity, as well as on the virus itself [6]. The degree of vitreous inflammation in NHR-affected eyes depends in part upon the underlying level of immune suppression, with trace-to-mild inflammation typically occurring in severely immuno-compromised patients with acquired immune deficit syndrome (AIDS), and moderate-to-severe inflammation occurring more often in patients with limited or no immune deficits [7]. It is now generally accepted that the presence or absence of overlying vitreous inflammation is determined largely by the patient’s underlying immune status [8].

In our case, the patient had comorbidities – a congenital cardiac anomaly, and PLE – and had long been using oral steroids. Immuno-suppressed patients are particularly at risk of developing necrotizing retinopathies typically associated with such states; for example, CMV retinitis and PORN. They may also develop syndromes that are more typically seen in immuno-competent hosts, such as ARN [9]. In the present case, the patient complained of only minimal discomfort and peripheral visual field defect at his initial visit, and conjunctival hyperemia and anterior chamber reaction were barely detected. That said, the funduscopic findings were more similar to those of ARN than to those of CMV retinitis. We resolved to administer IV ganciclovir (5 mg/kg, twice daily) and IV acyclovir (12 mg/kg, 3 times daily) simultaneously as an initial treatment. There were several reasons for this decision. Firstly, there was minimal inflammation in the anterior chamber and vitreous, and ARN typically presents a considerable amount of inflammation. Furthermore, the patient was considered immuno-compromised in that he suffered from PLE and had a history of long-term steroid use. More importantly, he was only 11 years old, and the disease was already alarmingly and surprisingly advanced at presentation.

Although PCR testing is not a decisive method for diagnosis of ARN, there have been several reports mentioning that the technique may be helpful in providing a more definitive diagnosis, and thus informing treatment [10]. After anterior chamber paracentesis, we were able using PCR to detect VZV DNA in the aqueous humor. Following this discovery, IV ganciclovir was stopped, and an increased dose of IV acyclovir was initiated; retinal lesions subsequently improved. We can conclude then, that if the patient shows atypical features at presentation, as occurred in this case, PCR testing is helpful.

Until recently, there were many reports addressing NHR or PORN in HIV-infected patients, but only a few in HIV-negative patients [6, 9, 11–13]. Specifically, the latter concerned patients after hematopoietic stem cell transplantation, kidney transplantation, and with transient immune deviation. To our knowledge, ours is the first described case of NHR in a PLE patient. The patient had received Fontan surgery in infancy and had subsequently developed PLE. For treatment and maintenance of his medical condition, he had been using oral steroids for 1 year and 2 months at presentation.

PLE can develop as a complication of the Fontan procedure for single-ventricle congenital heart disease. Some researchers have also reported immune abnormalities in Fontan-related PLE [14, 15]. For example, Magdo et al. recently concluded that Fontan-related PLE involves marked, quantitative, humoral and cell-mediated immune abnormalities, with severe CD4 lymphopenia [15].

Lim et al. reported a case of NHR that demonstrates well the change in clinical manifestations from one end of the spectrum to the other in the same patient, and that this change corresponds to the CD4 counts [11]. Considering these factors, even though we did not check our patient’s CD4 lymphocyte count (total lymphocyte count: 540 cells/μl), the clinical manifestations in this case may have been related to the patient’s medical condition. Despite the fact that every PLE patient studied has had very low CD4 counts, no opportunistic infections have been found – as may be expected with CD4 lymphopenia. Nonetheless, the debate continues regarding antibiotic prophylaxis, because there have not been enough patients or studies to draw conclusions [15]. Similarly, we cannot conclude that this patient’s immuno-compromised status was caused by his PLE, or by his long term steroid use. More studies addressing the immunological characteristics of PLE are indicated.

In summary, to our knowledge, ours is the only described case to date of NHR in a PLE patient. His immuno-suppressed status affected the degree of inflammation and made diagnosis difficult. This case suggests that meticulous retinal examination, combined with consideration of the patient’s immunological status, is important in NHR diagnosis.

## Abbreviations

ARN, acute retinal necrosis; CMV, cytomegalovirus; VZV, varicella-zoster virus; PCR, polymerase chain reaction; PLE, protein-losing enteropathy; BCVA, best-corrected visual acuity; HSV, herpes-simplex virus; PORN, progressive outer retinal necrosis; NHR, necrotizing herpetic retinopathy; AIDS, acquired immune deficit syndrome.

## References

[1] Duker JS, Blumenkranz MS (1991). Diagnosis and management of the acute retinal necrosis (ARN) syndrome. Surv Ophthalmol.

[2] Engstrom RE, Holland GN, Margolis TP, Muccioli C, Lindley JI, Belfort R, Holland SP, Johnston WH, Wolitz RA, Kreiger AE (1994). The progressive outer retinal necrosis syndrome. A variant of necrotizing herpetic retinopathy in patients with AIDS. Ophthalmology.

[3] Matsuo T, Nakayama T, Koyama T, Koyama M, Matsuo N (1988). A proposed mild type of acute retinal necrosis syndrome. Am J Ophthalmol.

[4] Holland GN (1994). Standard diagnostic criteria for the acute retinal necrosis syndrome. Executive Committee of the American Uveitis Society. Am J Ophthalmol.

[5] Guex-Crosier Y, Rochat C, Herbort CP (1997). Necrotizing herpetic retinopathies. A spectrum of herpes virus-induced diseases determined by the immune state of the host. Ocul Immunol Inflamm.

[6] Wensing B, de Groot-Mijnes JD, Rothova A (2011). Necrotizing and nonnecrotizing variants of herpetic uveitis with posterior segment involvement. Arch Ophthalmol.

[7] Cunningham ET, Wong RW, Takakura A, Downes KM, Zierhut M (2014). Necrotizing herpetic retinitis. Ocul Immunol Inflamm.

[8] Wong RW, Jumper JM, McDonald HR, Johnson RN, Fu A, Lujan BJ, Cunningham ET (2013). Emerging concepts in the management of acute retinal necrosis. Br J Ophthalmol.

[9] Chiang E, Pyatetsky D (2012). Acute retinal necrosis secondary to varicella zoster virus in an immunosuppressed post-kidney transplant patient. Clin Med Res.

[10] Park SS, Holz HA, Ravage ZB, Merrill PT, Nguyen QD (2008). Diagnostic and therapeutic challenges. Acute retinal necrosis syndrom. Retina.

[11] Lim WK, Chee SP, Nussenblatt RB (2005). Progression of varicella-zoster virus necrotizing retinopathy in an HIV-negative patient with transient immune deviation. Albrecht Von Graefes Arch Klin Exp Ophthalmol.

[12] Kalpoe JS, van Dehn CE, Bollemeijer JG, Vaessen N, Claas EC, Barge RM, Willemze R, Kroes AC, Beersma MF (2005). Varicella zoster virus (VZV)-related progressive outer retinal necrosis (PORN) after allogeneic stem cell transplantation. Bone Marrow Transplant.

[13] Turno-Krecicka A, Boratynska M, Tomczyk-Socha M, Mazanowska O (2015). Progressive outer retinal necrosis in immunocompromised kidney allograft recipient. Transpl Infect Dis.

[14] John AS, Johnson JA, Khan M, Driscoll DJ, Warnes CA, Cetta F (2014). Clinical outcomes and improved survival in patients with protein-losing enteropathy after the Fontan operation. J Am Coll Cardiol.

[15] Magdo HS, Stillwell TL, Greenhawt MJ, Stringer KA, Yu S, Fifer CG, Russell MW, Schumacher KR (2015). Immune abnormalities in fontan protein-losing enteropathy: a case-control study. J Pediatr.

